# Functional enhancement of chitosan and nanoparticles in cell culture, tissue engineering, and pharmaceutical applications

**DOI:** 10.3389/fphys.2012.00321

**Published:** 2012-08-21

**Authors:** Wenjuan Gao, James C. K. Lai, Solomon W. Leung

**Affiliations:** ^1^Department of Civil and Environmental Engineering, School of Engineering, Idaho State UniversityPocatello, ID, USA; ^2^Department of Biomedical and Pharmaceutical Sciences, College of Pharmacy, and Biomedical Research Institute, Idaho State UniversityPocatello, ID, USA; ^3^Department of Civil and Environmental Engineering, School of Engineering, and Biomedical Research Institute, Idaho State UniversityPocatello, ID, USA

**Keywords:** chitosan, nanoparticles, tissue engineering, biocompatibility, nanotoxicity

## Abstract

As a biomaterial, chitosan has been widely used in tissue engineering, wound healing, drug delivery, and other biomedical applications. It can be formulated in a variety of forms, such as powder, film, sphere, gel, and fiber. These features make chitosan an almost ideal biomaterial in cell culture applications, and cell cultures arguably constitute the most practical way to evaluate biocompatibility and biotoxicity. The advantages of cell cultures are that they can be performed under totally controlled environments, allow high throughput functional screening, and are less costly, as compared to other assessment methods. Chitosan can also be modified into multilayer composite by combining with other polymers and moieties to alter the properties of chitosan for particular biomedical applications. This review briefly depicts and discusses applications of chitosan and nanoparticles in cell culture, in particular, the effects of chitosan and nanoparticles on cell adhesion, cell survival, and the underlying molecular mechanisms: both stimulatory and inhibitory influences are discussed. Our aim is to update the current status of how nanoparticles can be utilized to modify the properties of chitosan to advance the art of tissue engineering by using cell cultures.

## Introduction

Chitosan, produced by deacetylation of chitin, is a presumed non-toxic and hydrophilic polysaccharide (Tomihata and Ikada, 1997; Fukuda et al., 2006). Commercially, chitin and chitosan are obtained from shellfish sources such as crabs and shrimps. Chitin was first discovered by the French scientist Henri Braconnot in 1811 and “modified chitin” was renamed “chitosan” by Hoppe-Seiler in 1894 (Winterowd and Sandford, 1995). Research on chitin and chitosan accelerated in the 1900s and recently hundreds of articles have been published on chitosan. Bioapplications of chitosan were probably more popularized in the last 25 years; chitosan is currently better known to be a dietary supplement to the public than its other biomedical applications. Because of its low cost, large-scale availability, anti-microbial activity, as well as biodegradation and biocompatibility (Khor and Lim, 2003), chitosan has been widely used by researchers as an important and promising biomaterial in tissue engineering (Dorj et al., 2012; Hu et al., 2012), wound healing (Charernsriwilaiwat et al., 2012; Wang et al., 2012), drug delivery (Chen et al., 2012a; Liang et al., 2012), and other biomedical applications (Amarnath et al., 2012; Kumar et al., 2012). Other important properties of chitosan include its compatibility to natural glycosaminoglycan (GAG) (Muzzarelli et al., 2005; Bhardwaj and Kundu, 2012) and that it can be molded into a variety of forms, such as powder, film, sphere, gel, and fiber. These compelling features are important qualities in cell culture. Many researchers have chosen chitosan or the combinations of chitosan with other materials to culture different cell types (Khor and Lim, 2003; Charernsriwilaiwat et al., 2012).

Recently adding nanoparticles in cell cultures for treatments and therapeutic uses is among the newest developments other than probing and imaging in nanotechnology and biotechnology. Nanotechnology, first appeared in the twentieth century, is an area of science devoted to the manipulation of atoms and molecules of materials in the nanometer range. Nanoparticles include all particles that possess at least one dimension that is less than 100 nm: the material origins of the particles can be organic, inorganic, metals, polymers, etc. Because of the wide-range of potential applications, nanotechnology has recently emerged as one of the most commercially viable technologies. Nanoparticles possess unique properties, more importantly, a large surface-to-volume ratio; thus, many of these particles possess high surface reactivity. These favorable properties are being exploited in many directions in science and technology, more so recently in biomedical applications.

Nanoparticles can enter the body through the lung and skin by absorption or through the gastrointestinal track via intake of food, drink, and medication. Due to the ubiquitous existence of nanoparticles and other nanomaterials, human exposure to these nanoparticles is inevitable. Nanoparticles can enter and affect different organs and tissues such as brain, liver, kidney, heart, blood, etc., and induce cytotoxic effects (Lai et al., 2008b). These particles may alter and inhibit cell growth leading to various pathophysiological states in humans and animals. Consequently, nanotoxicology research is now gaining much more attention and its importance is gradually being recognized.

Cell culture can be used to investigate cytotoxicity of nanoparticles including their effects on cell adhesion, cell survival, etc. Cell cultures *in vitro* can be performed under controlled environments with predictable and reproducible results, and are relatively inexpensive. Hence, research studies using nanoparticles in cell cultures have drawn considerable interest recently.

Combinations of material science in biological field proved to be fruitful and promise to hold great potential in biomedical developments: In 2004, Gu et al. (2004) induced proliferation of hepatocytes by immobilizing these cells on 24-nm gold colloids; they also constructed a silver nanocrystalline chitosan for wound dressing (Lu et al., 2008). In 2009, they found that the gold colloid/chitosan scaffold could promote adhesion and proliferation of keratinocytes (Zhang et al., 2009; Lu et al., 2010). Commercialization of these new technologies is sprouting almost as fast as they are developed, such as some of the wound dressing materials associated with nanoparticles (Rustogi et al., 2005; Ulkur et al., 2005).

In the biological and biomedical fields, studies involving chitosan, nanoparticles, or cell cultures alone are numerous in the literature. As the science of nanotechnology continues to advance, researchers are exploring different ways to modify chitosan for various biomedical applications. Moderate amount of work thus far has been performed to evaluate the biocompatibility of chitosan and modified chitosan in cell cultures, especially cultures of fibroblasts.

Applications of chitosan and its modified forms to tissue engineering is a fast developing field. This review highlights applications of chitosan and nanoparticles in cell cultures, in particular the effects of chitosan and nanoparticles on cell adhesion and cell survival: both stimulatory and inhibitory influences are discussed. Our aim is to update the current status of how nanoparticles can be utilized to modify the properties of chitosan to advance the art of tissue engineering (Lai et al., 2011) by using cell cultures.

## Uses of chitosan in cell cultures

### Deacetylation of chitosan

Many studies showed that the degree of deacetylation and variation in molecular weights are the two factors that determined the effects of chitosan on cell growth. Generally, chitosan dissolves in dilute acidic solutions such as HCl, acetic acid, and some other organic acids (Sashiwa et al., 2000). Howling et al. (2001) examined the effects of chitin and chitosan solution at various deacetylation levels (37%, 58%, and 89%), molecular weights (12,000–263,800 Da), as well as different concentrations (2.5–500 μg/mL) on the proliferation of human dermal fibroblasts and immortalized human keratinocytes (HaCaT) *in vitro*. Their study showed that at high degrees of deacetylation, chitosan stimulated fibroblast proliferation better than chitosan with lower levels of deacetylation; but the effects on keratinocytes were different. At high levels of deacetylation (89% deacetylated), chitosan inhibited HaCaT proliferation up to about 26%, while at lower degrees of deacetylation (37% deacetylated), chitosan had no effect on HaCaT proliferation at the reported concentrations. These findings indicated that the deacetylation level of chitosan is a key factor in regulating the mitogenic activity of fibroblasts and keratinocytes, but the cell responses with molecular weight differential was not clearly delineated in the report.

### Modifications of chitosan and its properties

Modification of chitosan either by substituting the surface functional groups or cross-linking chitosan with different layers of polymers can alter the mechanical and biological properties of chitosan. Chen et al. (2002) investigated the effects of carboxymethyl (CM)-chitosan at different concentrations (50–500 μg/mL) and different molecular weights (3,200 Da, 15,000 Da and 35,000 Da) on normal human skin fibroblast and keloid fibroblast. They showed that CM-chitosan promoted proliferation of normal skin fibroblast significantly but inhibited proliferation of keloid fibroblast, because the CM-chitosan could decrease the ratio of type I/III collagen in keloid fibroblast by suppressing the secretion of type I collagen; but CM-chitosan had no effect on the secretion of types I and III collagen in the normal skin fibroblast. While both high and low concentrations of the CM-chitosan promoted initial proliferation, they noted that at the high concentration (500 μg/mL), CM-chitosan exerted a more positive effect on initial cellular proliferation. On the other hand, at the lower concentration (100 μg/mL), CM-chitosan exerted a lower positive effect, compared to that of the higher concentration on initial cellular proliferation. Nevertheless, the duration of the effect exerted at the lower CM-chitosan concentration was longer than that achieved at the higher CM-chitosan concentration. In addition, molecular weight made no difference in growth stimulation for the skin fibroblast, the lower molecular weight (3,200 Da) CM-chitosan exerted almost the same effect on the growth stimulation of normal skin fibroblast as that achieved by the higher molecular weight (35,000 Da) CM-chitosan. However, the growth inhibition of keloid fibroblast increased slightly with the decrease of CM-chitosan molecular weight although that difference was not significant (~2%).

## Cell cultures on chitosan film

### Cell growth on chitosan film

Chitosan solution can be employed to produce chitosan film/membrane using solution-casting technique (Tomihata and Ikada, 1997). This film can serve as a scaffold for cell cultures. Fakhry et al. (2004) employed two kinds of commercially available chitosan of different deacetylation and molecular weights, Chitosan-H (molecular weight: 140,000 Da; degree of deacetylation: 80%) and Protasan CL212 (molecular weight: 270,000 Da; degree of deacetylation: 70%), to prepare chitosan film/membrane. They used these films to culture mouse osteoblasts and fibroblasts and found that the two chitosan films could enhance the initial attachment and proliferation of osteoblasts, but the films were not as effective with fibroblasts. Their observation indicated that manipulation of deacetylation and molecular size of chitosan could modulate the *in vitro* level of cell attachment and spreading. Indeed, chitosan film inhibited the proliferation and differentiation of embryonic rat cerebral cortical single stem cells and neurosphere in serum-free medium; but when serum was included, it induced the neurosphere-forming cells into an extensive cellular substratum of “protoplasmic cells” on which process-bearing cells spread (Hung et al., 2006). Zheng et al. (2003) studied the cytocompatibility of chitosan and CM-chitosan in human skin fibroblasts. They found that chitosan film inhibited cell growth and eventually the cells detached from the film. By contrast, the skin fibroblasts adhered to and differentiated well on CM-chitosan films. These findings demonstrated that cytocompatibility of CM-chitosan films in fibroblasts was better than that of plain chitosan films; these results were good examples that cell proliferation and differentiation were controlled by complex environmental conditions (stresses) that involve more than just manipulation of cell types alone.

Lahiji et al. (2000) hypothesized that chitosan promotes the survival and function of human osteoblasts and chondrocytes. Human osteoblasts propagating on chitosan films continued to express Type I collagen whereas chondrocytes expressed Type II collagen. Their results demonstrated the biocompatibility of chitosan as a substrate for the growth and continued function of human osteoblasts and chondrocytes. Consequently, chitosan shows some potential as a tissue engineering tool for the repair of osseous and chondral defects.

The effects of hexanoyl chitosan (H-chitosan) on cytotoxicity, attachment, proliferation, and spreading of L929 (mouse fibroblast) cells were reported (Neamnark et al., 2007). The attachment of the cells on H-chitosan film was better than that on the chitosan counterpart for a short time (<5 h) after seeding, while the proliferation of the cells on H-chitosan film was better than that on the chitosan counterpart after 2 and 3 days in culture.

To simulate a membrane layer that is closer to the natural membrane that can be used as a structure scaffold and function as a barrier with selectivity, a single layer material for such is nearly impossible but a bilayer membrane is more realistic. A bilayer structure of chitosan film and sponge as a scaffold can support growth and proliferation of human neofetal dermal fibroblasts (Ma et al., 2001). In fact, blending materials with different chemical and physical properties (biocomposites) are finding numerous applications in biomedical technology.

### Blending polymers with chitosan as cell scaffold

To improve the biocompatibility and other properties (e.g., permeability) critical for a wide-range of biomedical applications, blending chitosan with other polymers has been widely investigated. Silica-chitosan complex membrane (SiCM) was developed by Suzuki et al. (1999). The composition of the SiCM was stepwisely controlled by adjustments of mixing ratios between silica and chitosan from 0% to 50%. Their study showed that 50% SiCM was especially effective in increasing adhesion and growth of human embryonic kidney cells and human lung diploid cells. Bettini et al. (2008) used sugar and phosphate to modify the chitosan film: phosphate and sugar were added into the chitosan film-forming solutions. The resulting chitosan film exerted a beneficial effect on affinity with human diploid fibroblasts (HDF, cell strain WI-38). A novel absorbable scaffold with an asymmetric structure composed of chitosan and gelatin was fabricated by a freezing and lyophilizing method as a bilayer skin substitute (Mao et al., 2003). Data from that study suggested that scaffold constructed by this artificial bilayer was flexible, had good mechanical properties and induced no contraction in the cell cultures tested. Using the suspension of chitosan hydrogel mixed with gelatin, novel chitosan/gelatin membranes were prepared (Nagahama et al., 2009); human MG-63 osteoblast-like cells were used to examine the effect of chitosan/gelatin membranes on cell adhesion. The cells incubated with chitosan/gelatin membranes for 24 h exhibited good adhesion to the chitosan/gelatin substratum, suggesting that these modified chitosan membranes are useful for biomedical applications. Huang et al. (2005) investigated the degradation of chitosan-gelatin scaffold and its effect on mouse fibroblast activity and adhesion. The degradation rate and material loss of chitosan-gelatin scaffold were faster than those of chitosan alone even though both chitosan-gelatin and chitosan alone supported fibroblast viability equally well. A similar study was also performed by Chupa et al. (2000): they evaluated the potential applications of GAG-chitosan and dextran sulfate (DS, a semi-synthetic GAG analog)-chitosan complex membrane materials for controlling the proliferation of human vascular endothelial (EC) and smooth muscle cells (SMC). Their results showed that while chitosan alone supported cell attachment and growth, GAG-chitosan materials inhibited spreading and proliferation of ECs and SMCs *in vitro*. In contrast to the GAG-chitosan, DS-chitosan supported proliferation of both cell types, although it is a semi-synthetic GAG analog. Their results indicated that GAG-chitosan could also be used to modulate the proliferation of EC cells. A series of chitosan/poly-l-lysine composite films were produced from chitosan/poly-l-lysine blended solutions by Zheng et al. (2009) The effects of the composite films on the behavior of MC3T3-E1 osteoblast-like cells (from mouse) were assessed. Those researchers found that the increase in poly-l-lysine weight fraction in the blended solutions could result in different nanoscaled surface topographic features, and MC3T3-E1 cells strongly responded to those nanotopographic features. Consequently, their novel observation demonstrated nanotopography of chitosan and chitosan-derived materials could exert remarkable influences on behavior of cells cultured *in vitro*. Thus, topographic modification of chitosan-derived substratum on a nanoscale may be exploited in regulating behavior of cells in culture, and as such can be productively exploited in diverse applications in tissue engineering, as well as in engineering an environment to foster bone regeneration.

Elastin and poly-l-lysine were used to modify a hybrid bulk scaffold which contained polyethylene oxide, chitin, and chitosan (Kuo and Chung, 2012). Bovine knee chondrocytes were seeded in the scaffolds and cultured in a spinner-flask bioreactor over 4 weeks. Results of this study showed that elastin- and poly-l-lysine-grafted polyethylene oxide/chitin/chitosan scaffolds were effective in producing cartilaginous components. It appears that modified chitosans have more and better control of biomedical applications than just chitosan alone due to better physicochemical properties.

### Complex composite modifications of chitosan film

Once chitosan is viewed as a building component of composite materials for biomedical applications, the possibilities of creating new biocomposites with chitosan are almost unlimited. With such, majority of the new applications thus far are associated with improving structural properties of scaffolding and tissue connection. The following are some of the major applications of chitosan biocomposites.

#### Applications with bone/cartilage

This constitutes another strategy to develop novel tissue engineering applications. For example, Chen et al. (2006) utilized fractional factorial design methodology to co-immobilized four different GAGs (chondroitin-4-sulfate, chondroitin-6-sulfate dermatan sulfate and heparin) and prepared eight different combinations of GAG/chitosan membranes. They evaluated the effects of these GAG/chitosan membranes on several properties of chondrocytes, including adhesion, morphology, and proliferation. This was their attempt to provide a rational method to predict and evaluate the proper formulation of GAG/chitosan membranes for various applications in tissue engineering. Recently, chitosan/chondroitin sulfate/nano-SiO_2_ composite scaffold was fabricated by lyophilization (Kavya et al., 2012). Biocompatibility and cell attachment-proliferation studies performed using MG-63 cells (human osteosarcoma cell line) suggested this novel nanocomposite scaffold could be a suitable candidate for bone tissue engineering.

The polypeptide Arg-Gly-Asp-Ser (RGDS) sequence of fibronectin was used to modify chitosan membrane using the photochemical immobilization technique (Karakecili et al., 2007): this substrate could enhance the attachment and proliferation of L929 (mouse fibroblast) cells. Schneider et al. (2007) cross-linked chitosan with hyaluronan (a polysaccharide) (CHI/HA) using a water-soluble carbodiimide: the film had a much improved elastic modulus and was more resistant to enzymatic degradation by hyaluronidase but was not overly thick. Consequently, the increased film stiffness improved the adhesion and spreading of chondrosarcoma cells. Thus, the CHI/HA cross-linked films could be used for various biomedical applications due to their more favorable mechanical and adhesive properties, and are a more robust matrix material due to their higher tolerance to enzymatic degradation. Higher resistance to enzymatic degradation allows the engineered tissue to develop into more mature structure before the supportive scaffold collapsed. A series of nano-hydroxyapatite/chitosan cross-linking composite membranes (nano-hydroxyapatite; 0–30% by weight) were successfully developed by a simple casting/solvent evaporation method by Li et al. (2012). The nano-hydroxyapatite content greatly affected the morphology as well as the tensile property of composite membrane. *In vitro* cytotoxicity testing suggested that the developed nano-hydroxyapatite/chitosan cross-linking composite membrane was non-cytotoxic to L929 cells after 24 h of incubation. Therefore, the nano-hydroxyapatite/chitosan cross-linking composite membrane with favorable cytocompatibility, water adsorption (wettability) and tensile strength might serve as the vehicle for bone tissue engineering.

Chitosan has also been employed to reinforce calcium phosphate cement (CPC). Moreau and Xu (2009) examined the proliferation and osteogenic differentiation of mesenchymal stem cells (MSCs) cultured on high-strength CPC-chitosan scaffold. On the CPC-chitosan scaffold, MSCs differentiated into the osteogenic lineage and expressed high levels of bone marker alkaline phosphatase (ALP). The results of Moreau and Xu (2009) suggested the stronger CPC-chitosan scaffold may be useful for tissue engineering research in stem cell-based bone regeneration.

Alginate-chitosan semi-interpenetrating polymer network (IPN) scaffolds were prepared by a freeze-drying process (Tigli and Gumusderelioglu, 2009). The attachment and proliferation abilities of ATDC5 murine chondrogenic cells on alginate, 70:30% (v/v) alginate:chitosan and 50:50% (v/v) alginate:chitosan scaffolds, were assessed. The results of this study indicated that alginate:chitosan semi-IPN scaffolds could promote chondrocyte proliferation and that 50:50% (v/v) alginate:chitosan scaffolds showed some promise as an ideal material for applications in cartilage tissue engineering *in vitro*, especially from the prospective of structural analysis and cell-based functional screening.

Venkatesan et al. (2012) constructed chitosan-carbon nanotube scaffolds by a freeze-drying method. Carbon nanotube was uniformly dispersed in chitosan matrix. Cytotoxic effects and cell proliferation of scaffold were investigated employing the MTT assay using MG-63 cells (human osteosarcoma cell line). The cell proliferation, protein content, ALP and mineralization of the cells cultured on composite scaffolds were higher than those on the chitosan scaffold due to the addition of carbon nanotube suggesting that chitosan/f-MWCNT scaffolds may be promising biomaterials for bone tissue engineering.

#### Applications with fibroblasts and other cell types

Chitosan can also be used to functionalize with other polymerizable molecules to improve the biocompatibility and mechanical properties of the resulting copolymer, such as poly-l-lactic acid, poly(L-lactide-co-epsilon-caprolactone) and poly (ε-caprolactone) (Ding et al., 2004; Mei et al., 2005; Jiao et al., 2007; Yang et al., 2012; Zhang and Cui, 2012). Immobilization of chitosan onto poly-l-lactic acid (PLLA) film surface by plasma graft polymerization has been reported. Two cell lines, L929 (mouse fibroblast) and L102 (human hepatocytes), were cultured on the modified PLLA surface. Results indicated that cell spreading on this film was minimal and the colonies tended to become rounded. Thus, the film was demonstrated to be a poor adhering substrate. Nonetheless, cells grown on this substrate proliferated at almost the same speed as those cultured on a glass surface. These results suggested that the new substrate could be used to control the morphology of cells.

Another method to construct a novel cytocompatible graft co-polymer of chitosan and L-lactic acid was reported by Yao et al. (2003). They dissolved chitosan powder in an aqueous solution of l-lactic acid and then poured the solution into a frame mold, and maintained at a proper temperature for film formation. With this type of co-polymer films, they were able to ascertain that the growth rate of human fibroblast on the co-polymer films, with various l-lactic acids to chitosan ratios, was higher than that on chitosan alone, but the growth of those cells decreased as the ratio of l-lactic acid to chitosan increased. All the copolymer and chitosan films enhanced initial fibroblast proliferation, but the proliferation of the cells on both types of films eventually became slower than that of the controlled cells cultured after 9 days. Evidently, despite their results of slower cell growth at prolonged time, the co-polymer films provided a better scaffold than chitosan alone with improved mechanical properties for cell growth and thus could be useful in applications in designing grafting in tissue engineering.

Keratin-chitosan film is another useful composite film fabricated (Tanabe et al., 2002). This film was fabricated by mixing solutions of keratin and chitosan casting into the polypropylene mold, and then followed by drying at 50°C overnight. With this procedure, films with average 0.01–0.02 mm thickness were obtained. The role of chitosan was to reinforce the mechanical properties of keratin film. Results of that study (Tanabe et al., 2002) showed the keratin-chitosan film was a good substrate that supported the attachment and proliferation of L929 mouse fibroblast cells; this finding also suggested that keratin-chitosan film could be a good supporting substrate for other mammalian cells in culture.

Poly (vinyl alcohol) (PVA)/chitosan blend has also been fabricated (Chuang et al., 1999). Human skin fibroblasts seeded and cultured on the PVA/chitosan blended membrane showed better attachment and spreading compared to that of fibroblasts seeded and cultured on the pure PVA membrane alone (Chuang et al., 1999). This observation suggested that chitosan enhanced the biocompatibility of the PVA and that the PVA/chitosan blended membrane could be employed as another useful biofilm for cell culture applications. In 2009, Costa et al. (2009) and Mansur et al. (2009) synthesized chitosan/PVA blends with different chitosan/PVA mass ratios and chemically cross-linked these blends with glutaraldehyde. VERO cells (isolated from kidney epithelial cells from an African green monkey) were used to assess the biocompatibility and cytotoxicity of these chitosan/PVA blends. Their studies demonstrated that changing the mass ratio of chitosan to PVA could alter the “swelling behavior” of the blended materials. Swelling control is an important property for tissue engineering that the physical performance of the materials must be predictable. The results from cell biocompatibility assays have demonstrated that all these blends were non-toxic and biotolerant and potentially suitable for prospective use in skin tissue engineering and drug delivery.

## Three-dimensional cell culture matrices involving chitosan gel and chitosan nanoparticles/nanofiber complexes

### Cell growth in 3-dimensional chitosan structures

Under physiological conditions, cells normally proliferate and grow in various organs and tissues *in vivo* in a truly three-dimensional (3-D) matrix surrounded by one or more other cell types (Lai and Leung, 2005; Zhang et al., 2005). Thus, a 3-D *in vitro* cell culture environment more closely resembles that of the *in vivo* physiological environment. Methods or materials fare well in the 2-D setting may not fare well in the 3-D situation; one of the major obstacles of 3-D culture is the delivery or availability of nutrients that are needed for the cells to survive/proliferate. Passive diffusion does not provide enough supports for the cells in deep layers: this situation may not be as critical in 2-D culture. Indeed, the use of chitosan has been recently exploited to create such an environment *in vitro*. For example, chitosan can be made into a gel and/or nanoparticle/nanofiber complex that can provide a 3-D structure. Poly (vinyl alcohol) (PVA)/chitosan blended hydrogel has been fabricated for that purpose (Koyano et al., 1998; Minoura et al., 1998). The hydrogel with 40% (weight) chitosan content was superior to collagen for the attachment and growth of L929 mouse fibroblasts. Karp et al. (2006) created micropatterns in the chitosan gel using a photolithographic method which was simple and rapid. Cardiac fibroblasts, cardiomyocytes and osteoblasts could form arrays on these chitosan-patterned surfaces and remained stable for up to 18 days. Zheng et al. (2012) recently presented a new method for primary neonatal rat cardiomyocytes cultured *in vitro* using alginate/collagen/chitosan hydrogel. The results showed a significant increase in cardiac myocyte numbers, and the expression levels of CACNL1A1 and Connexin 43 were up-regulated significantly, as compared with those in 2-D cultures. By using a electrospinning technique, PCL (poly(ε-caprolactone))/chitosan/PCL scaffolds were prepared layer by layer (Sasmazel, 2011). The hybrid scaffolds exhibited nanofiber structures and such layered scaffolds have provided improved substrate delivery to facilitate the growth and differentiation of SaOs-2 osteosarcoma cells cultured *in vitro*. A biomimetic poly(propylene carbonate) (PPC) porous scaffold with nanofibrous chitosan network (PPC/CSNFs, chitosan nanofibers) for bone tissue engineering was fabricated by a dual solid-liquid phase separation technique (Zhao et al., 2012). The *in vitro* culture of bone MSCs showed that PPC/CSNFs scaffold exhibited a better cell viability than PPC scaffold. Bhardwaj and Kundu (2012) fabricated polyelectrolyte complex silk fibroin/chitosan blended porous scaffolds and examined its ability to support *in vitro* chondrogenesis of MSCs. These results suggested that silk fibroin/chitosan blended 3D scaffolds were suitable scaffold for mesenchymal stem cell-based cartilage repair. A similar study showed that silk fibroin/chitosan composite nanofibers could support the growth and osteogenic differentiation of human fetal osteoblastic cells, indicating that these nanofibers would be potentially suitable for bone tissue engineering applications (Chen et al., 2012b).

Fukuda et al. (2006) advanced the use of chitosan hydrogel in the co-culture paradigm for cells from different animal species. They synthesized a photo-cross-linkable chitosan using the protocol of Ono et al. (2000). Fukuda and co-workers demonstrated human hepatocellular carcinoma (HepG2) cells and NIH-3T3 mouse fibroblasts could be co-cultured in the chitosan hydrogel where the two cell types were spatially separated. They also noted that the NIH-3T3 fibroblasts attached evenly to the chitosan surface surrounding the HepG2 spheroids and proliferated over time to cover the entire surface of the hydrogel.

Manipulating neural cells in culture has consistently provided challenges for tissue engineering researchers, not least of all because of the paucity of existing appropriate neural cell models *in vitro* (Lai and Leung, 2005; Zhang et al., 2005). On the other hand, model systems *in vivo* have revealed significant challenges that differ from those of *in vitro* models (Lai and Leung, 2005; Zhang et al., 2005). Nevertheless, some advances have been evident. Zahir et al. (2008) investigated the survival and differentiation of neural stem/progenitor (NSPCs) cells cultured on chitosan matrices *in vivo* in a complete transection model of spinal cord injury. Firstly, they isolated NSPCs from the subependyma of lateral ventricles of adult green fluorescent protein (GFP)-transgenic rat forebrains, and then seeded the GFP-positive neurospheres onto the inner lumen of chitosan tubes to generate multicellular sheets *ex vivo*. Finally, they implanted the bioengineered neurosphere tubes into a completely transected spinal cord for 5 weeks and then assessed for cell survival and differentiation. They found that the implanted NSPCs showed excellent survival; moreover, the implanted cells differentiated into astrocytes and oligodendrocytes. These results demonstrated that the chitosan tubes exhibited excellent potential for use in stem cell delivery and neural regeneration. For neural tissue engineering, hydrogel-based scaffolds can provide appropriate physicochemical and mechanical properties to support neurite extension. Valmikinathan et al. (2012) developed a novel chitosan-based photocrosslinkable hydrogel system with tunable mechanical properties and degradation rates. When human MSCs were cultured in photocrosslinkable chitosan hydrogels, negligible cytotoxicity was observed. Photocrosslinkable chitosan hydrogels facilitated neural differentiation from primary cortical neurons and enhanced neurite extension from dorsal root ganglia as compared to agarose based hydrogels. These data demonstrated the potential of photocrosslinked chitosan hydrogels designed for neural tissue engineering. Electrospun polyvinyl alcohol (PVA)/chitosan nanofibrous scaffolds have been synthesized (Alhosseini et al., 2012). The scaffolds were used for *in vitro* cell culture in contact with PC12 cells which are neurons-like, they were found to exhibit the most balanced physicochemical and biological properties to meet the basic required specifications for nerve cells. Thus, it could be concluded that addition of chitosan to the PVA scaffolds enhanced viability and proliferation of nerve cells, thereby confirming the biocompatibility of the scaffolds. Therefore, PVA/chitosan nanofibrous scaffolds have the potential to be used in nervous tissue engineering and repair.

The thermosensitive chitosan-gelatin-glycerol phosphate hydrogels were synthesized so that they can be employed as a cell carrier for nucleus pulposus (NP) cell regeneration (Cheng et al., 2010). NP cells cultured in the hydrogels displayed normal GAG production, mRNA production and gene expression. This finding suggested that this hydrogel may be suitable for intervertebral disc replacement in tissue engineering. Ma et al. (2010) prepared injectable hydrogels from chitosan derivative, methacryloyloxy ethyl carboxyethyl chitosan (EGAMA-CS)/polyethylene glycol dimethacrylate (PEGDA)/N, N-dimethylacrylamide (DMMA) by photopolymerization. Their cell culture studies demonstrated that these hydrogels exhibited the desirable properties in promoting attachment and proliferation of human bone sarcoma (SW1353) cells. Thus, their results suggested that these hydrogels may well be the ideal matrix material for bone tissue engineering. More recently, porous chitosan-gelatin/hydroxyapatite composite scaffolds were developed (Isikli et al., 2012). These scaffolds promoted Saos-2 cells attachment and proliferation indicating that scaffolds prepared from chitosan, gelatin and hydroxyapatite were good cell carriers for bone tissue engineering.

An important issue to tissue engineering is the influence of mechanical characteristics and matrix architecture of substrates used in cell culture. Iyer et al. (2012) examined the influence of porous structures, hydrogels (chitosan-gelatin), and membranes of chitosan-based material on the growth of normal human fibroblasts and their matrix production in a serum-free system. They used chitosan alone and in combination with gelatin. They found that increased viability of fibroblasts on chitosan gelatin porous scaffold with decreased proliferation relative to tissue culture plastic surface. The total protein, collagen and tropoelastin contents were higher in the spent media derived from cells cultured with chitosan gelatin porous scaffolds compared with corresponding levels from cells treated with chitosan membrane or hydrogel alone. An increase in collagen content was also observed in the matrix, suggesting increased matrix deposition. Therefore, matrix production is influenced by the form of chitosan structures, which significantly affects the regenerative process.

### Effects of chitosan nanoparticles on cells in culture

Chitosan nanoparticles have been synthesized using either the batch processing methods or the spinning disc processor (Bodna et al., 2005; Loh et al., 2008). Evidence has shown that chitosan nanoparticles may exert differential bactericidal and pharmacological effects on prokaryotic and eukaryotic cells in culture (Shi et al., 2006; Grenha et al., 2007). Shi et al. showed that chitosan nanoparticles had significant bactericidal effects on bacteria such as *Staphylococcus aureus* (*S. aureus*) and *Staphylococcus epidermidis* (*S. epidermidis*), but had no cytotoxic effect on mouse fibroblast cells (Shi et al., 2006). Grenha et al. also demonstrated that chitosan nanoparticles are biocompatible with human respiratory epithelial cells *in vitro* (Grenha et al., 2007). Nevertheless, chitosan nanoparticles cannot be expected to be biocompatible to all mammalian cell types. Materials that are non-toxic at macroscale can become toxic at nanoscale (Rustogi et al., 2005). Nafee et al. (2009) investigated the effects of the chitosan/PLGA[Poly(D,L-lactide-co-glycolide)] nanoparticles on three cell lines (i.e., African green monkey kidney COS-1 cells, human alveolar cancer A549 cells and human bronchial epithelial Calu-3 cells). These chitosan nanoparticles were cytotoxic to COS-1 cells in a dose-related manner; however, they were not cytotoxic to A549 cells in the dose range investigated. Similarly, these chitosan nanoparticles were nearly non-cytotoxic to Calu-3 cells compared to COS-1 cells. Similar results were obtained by employing different cytotoxic assays (e.g., MTT, LDH release) by the investigators.

### Effects of collagen-chitosan nanofibers

A few studies have focused on elucidating the effects of another novel chitosan nanofiber, namely collagen-chitosan complex nanofibers, which have been fabricated employing an eletrospinning technique (Chen et al., 2007). Tangsadthakun et al. (2007) investigated the influence of molecular weight of chitosan on the physical and biological properties of collagen/chitosan scaffolds. They found that low-molecular-weight chitosan within the collagen-chitosan nanofibers rendered the nanofibers more effective in promoting and accelerating the proliferation of mouse L929 fibroblasts. Duan et al. (2006) constructed a nanofibrous composite membrane of poly (lactide-co-glycolide) (PLGA) and chitosan/poly(vinyl alcohol) (PVA) and tested their effects on attachment and proliferation of rabbit dermal fibroblasts. They concluded that the nanofibrous composite membrane they fabricated show a good tendency toward promoting the attachment and proliferation of rabbit dermal fibroblasts and suggested that the composite membrane was a good candidate for application in skin tissue engineering/reconstruction. Feng et al. (2009) developed a novel natural nanofibrous galactosylated chitosan (GC) scaffold and investigated its effect on primary cultures of rat hepatocytes. They observed that hepatocytes cultured on GC nanofibrous scaffold formed stably immobilized 3-D flat aggregates and exhibited superior bioactivity with higher levels of liver-specific functions compared to the 3-D hepatocyte spheroid aggregates formed on GC films alone. Consequently, their findings pointed to the utility of the GC-based nanofibrous scaffolds in the constructs of bioartificial liver-support devices and the versatility of these scaffolds were also suitable as substrates for primary cultures of hepatocytes in tissue engineering applications, such as liver regeneration and related translational research.

## Nanoparticles and cell survival

### Toxicity of nanoparticles and related nanomaterials

Despite the important and accelerating advances of nanoscience and nanotechnology, there has been increasing concern that some of the nanomaterials are not as harmless as people have assumed. Those concerns have spurred a new field of research, namely, “nanotoxicology” (Jandhyam et al., 2008; Lai et al., 2008c).

Cell cultures of a variety of human and other mammalian cell types constitute versatile model systems *in vitro* for high throughput screening of putative toxicity of nanomaterials, including nanoparticles, and for elucidating any underlying molecular mechanisms (Rustogi et al., 2005; Jandhyam et al., 2008; Lai et al., 2008c). The putative nanotoxicity of various nanomaterials has been examined: such nanomaterials included, but were not limited to, carbon nanotubes (Agharkar et al., 2008), carbon nanopipes (Whitby et al., 2008), silicon dioxide (Rustogi et al., 2005) and many metal oxides.

In 2005, Limbach et al. (2005) evaluated the uptake and transport of industrially important cerium oxide nanoparticles into human lung fibroblasts *in vitro* by exposing them to suspensions of these nanoparticles, with concentrations ranging from 100 ppb to 100 ppm. At such low but pathophysiologically relevant concentrations, the size of the nanoparticles was a dominant factor in determining the rate of uptake, while total particle surface area was of secondary importance. In 2006, the same research group employed a human mesothelioma cell line and a rodent fibroblast cell line to test the putative cytotoxicity *in vitro* of seven industrially important soluble and insoluble nanoparticles, namely SiO_2_, Ca_3_ (PO_4_)_2_, Fe_2_O_3_, ZnO, CeO_2_, TiO_2_, and ZrO_2_. They found that solubility was the critical factor in determining the cytotoxicity of the nanoparticles (Brunner et al., 2006). In 2011, Shavandi et al. (2011) assessed the toxicity of silver nanoparticles in murine peritoneal macrophages. A significant decrease in cell viability was observed at concentration of silver nanoparticles from 1 ppm to 25 ppm when the results were compared to that in the control group after 24 h of exposure of the cells to the nanoparticles in culture.

Lai and co-workers were the first to develop cell models *in vitro* for high throughput screening of putative cytotoxicity of nanomaterials, including nanoparticles, in neural cells (Rustogi et al., 2005; Lai et al., 2007, 2008a,c, 2009, 2010; Agharkar et al., 2008; Jandhyam et al., 2008; Jaiswal et al., 2010, 2011; Jain et al., 2011; Lu et al., 2011). Their comprehensive and systematic studies have revealed that nanoparticles of metallic and non-metallic oxides exerted differential cytotoxic effects on neural cells derived from the central nervous system as cell models *in vitro* (Rustogi et al., 2005; Lai et al., 2007, 2008a,c, 2009, 2010; Jandhyam et al., 2008). More recently, they have also developed non-tumor neural cells derived from the peripheral nervous system for high throughput screening of putative cytotoxicity of nanomaterials, including nanoparticles, on peripheral neural cells (Jaiswal et al., 2010, 2011; Jain et al., 2011; Lu et al., 2011). Results of their previous and ongoing studies have also indicated that nanoparticles of metallic and non-metallic oxides exerted differential cytotoxic effects on neural cells derived from the peripheral nervous system (Jaiswal et al., 2010, 2011; Jain et al., 2011; Lu et al., 2011). Their results (Rustogi et al., 2005; Lai et al., 2007, 2008a,c, 2009, 2010; Jain et al., 2011; Jandhyam et al., 2008; Jaiswal et al., 2010, 2011; Lu et al., 2011) have highlighted the importance and need for screening and elucidating the putative cytotoxicity of nanoparticles in neural cells if the nanoparticles are to be employed in biomedical and other applications that exert impact on the nervous system. Indeed, one emerging area where this nanotoxicity concern needs to be adequately addressed is the area of targeted drug delivery to the central nervous system and other peripheral organs.

To enhance targeted drug delivery, nanoparticle-loaded polymer capsules are often employed to enable selective drug delivery to the desired target organ(s). In accord with the considerations discussed above, prior to—at least along with—developing the applications of nanoparticles for drug delivery, the putative nanotoxicity of such particles needs to be assessed. Some examples of this kind of investigations are available in the literature. Kirchner et al. (2005a) demonstrated that polymer capsules containing coating of CdTe nanoparticles exhibited higher toxic effects than those of non-coated capsules. Nevertheless, by comparison, the silica-coated CdSe/ZnS nanoparticles were far less cytotoxic (Kirchner et al., 2005b). On the other hand, not all nanoparticles containing Si are benign and non-toxic. Di Pasqua et al. (2008) assessed the cytotoxicity of MCM-41, a mesoporous silica nanomaterial, in human neuroblastoma SK-N-SH cells. They found that MCM-41 was more toxic than spherical silica (i.e., SiO_2_) nanoparticles. As we have alluded to above, SiO_2_ nanoparticles are known to be cytotoxic to neural and non-neural cells (Rustogi et al., 2005; Lai et al., 2007, 2008a, 2010; Jandhyam et al., 2008; Jain et al., 2011; Jaiswal et al., 2011).

### Applications of nanoparticles

Cyclodextrins (CDs) are cyclic oligosaccharides widely used in pharmaceutical industries to control and/or improve on the solubility, release and absorption of drugs so as to ascertain the proper delivery of such drugs. Thus, injectable CDs are useful in drug delivery applications. Their utility notwithstanding, one disadvantage of using CDs is that the CDs can induce hemolysis and nephrotoxicity. Consequently, there is a need to obviate such adverse effects induced by the CDs: in this area, research is still continuing. For example, concentration-related cytotoxicity of β-CDC6, an amphiphilic β-cyclodextrin derivatized nanoparticles, has been demonstrated by using mouse fibroblasts and human polymorphonuclear cells in culture (Memisoglu-Bilensoy et al., 2006).

Among the metal nanoparticles, nano-gold particles possess favorable biocompatibility and stability (Gu et al., 2001, 2002, 2003, 2006). The utility of nano-gold particles was first highlighted in the immune-gold staining procedures in electron microscopic applications in the 1970s. But studies on the effects of the nano-gold particles on proliferation of mammalian cells only began to emerge in recent years (Lai et al., 2008b). In 2005, Mukherjee et al. (2005) noted that a novel mechanism by which nano-gold particles inhibited the proliferation of human umbilical EC cells *in vitro* was via binding with heparin-binding proteins. However, the toxicity of the nano-gold particles could be decreased by functionalizing with peptide of the sequence Gly-Arg-Gly-Asp-Ser-Pro (GRGDSP) (Fuente et al., 2006).

Ramis-Castelltort et al. (2008) designed a nanostructured material composed of a gold nanoparticle core functionalized with hyaluronan (HA) molecules on its surface. They tested this conjugate based on Gold-HA nanoparticles for cosmetic applications with the intent to improve features including stability, skin-penetration and water absorption/retaining effect, but their results remained to be confirmed. In order to improve the biocompatibility of polyethylene terephthalate (PET) mesh, scaffolds composed of PET-gold nanoparticles (PET-AuNP) were developed through conjugating various concentrations of AuNP to the PET surface (Whelove et al., 2011). Their results demonstrated that exposure of L929 murine fibroblasts to the PET-AuNP scaffolds improved their cell integrity, decreased their production of reactive oxygen species and lowered bacterial adhesion to the PET; thus, their findings suggested AuNPs could enhance the biocompatibility of the PET mesh. Kumari and Singh (2012) synthesized gold nanoflowers (flower-like, three dimensional branched gold nanoparticles) and constructed glycolic acid-g-chitosan-gold nanoflower nanocomposite scaffold. The nanohybrid scaffold was stable at the medium pH and it was biocompatible through cell (SP2/0 mouse myeloma cell line) viability study. These Au nanoflowers released the carried drug (cyclophosphamide) at rates depending on the depth (location) of the nanoparticles in buffer solution (pH 7.4). Therefore, these gold nanoflowers might be a viable additive for drug delivery for the glycolic acid grafted chitosan-based system, but the drug released mechanism *in vivo* was not confirmed.

Nano-gold particles can also be used for assay development. A nanoparticle-based antimicrobial susceptibility assay, utilizing the concanavalin A-induced clustering of dextran-coated gold nanoparticles, was developed (Perez et al., 2008). This gold nanoparticles-based assay reportedly provided reliable and faster results within 3 h, as compared to conventional methods that took 24–48 h. Liu et al. (2008) reported a one-step biomolecular detection method using gold nanoparticle bioconjugates. This detection method not only permitted the analysis of cancer protein biomarkers but also potentially allowed the development of bioassays for fast detection and quantitative analysis of DNAs, therapeutic drugs and other biological targets.

Silver nanoparticles have been widely used in cosmetic and medical applications. For example, silver nanoparticles have been used as an additive to burn-dressing. Depending on the methods by which they are produced, the physical and chemical properties of the nanoparticles may significantly differ. Ji et al. (2008) investigated the effects of two types of silver nanoparticles, those of colloidal silver and plasma silver, in human periodontal ligament cells. Consistent with the hypothesis stated above was their finding that the colloidal silver nanoparticles showed higher toxicity than plasma-generated silver nanoparticles, especially in inhibiting proliferation of the ligament cells. Another biomedical application of silver nanoparticles relates to their use in ameliorating joint inflammation. Use of the ointment containing silver nanoparticles led to faster recovery from temporomandibular joint arthritis (Lee et al., 2008); this beneficial effect was attributed to the anti-inflammatory action of the silver nanoparticles.

In summary, many nanoparticles appear to show some toxicity in various cell types. Consequently, in considering the use of nanoparticles in pharmaceutical and other biomedical applications, one should eliminate the putative cytotoxicity of such particles, which very often exert as a complicating and undesirable characteristic. On the other hand, one can productively exploit the toxicity of nanoparticles in conjunction with existing anti-cancer drugs to arrive at improved drug formulations of combination therapies to enhance their potential and efficacy for treating different kinds of cancers. All in all, the literature suggests that the strategic use of combinations of nanomaterials with other materials may lead to the development of more biocompatible materials with the desired characteristics for different pharmaceutical and other biomedical applications.

## Applications of chitosan and nanoparticles in bioengineering

A recent advance in nanotechnology is the development of a functional nanosystem (such as carbon nanotubes, quantum dots, polymeric micelles, metallic nanoparticles and liposomes), by incorporation, adsorption, or covalent coupling of polymers, carbohydrates, nucleic acids or polysaccharides to the surface of nanoparticles (Nahar et al., 2006). Various types of functional nanosystems are being extensively explored for diverse applications in the biomedical field. A major problem in tissue engineering is high tissue accumulation of non-biodegradable nanoparticles that may hinder mobility and cause inflammatory/toxicity problems, thus rendering them as “not-so-popular and not-so-desirable” therapeutic and diagnostic systems. This pathophysiological condition is presumed to be related to the immune response elicited by the non-biodegradable nanoparticles and evidently needs further research.

In bioengineering, the layer-by-layer technique used to build thin polyelectrolyte multilayer films is a new promising approach to modify biomaterial surfaces (Karakecili et al., 2007). Adopting this approach, one can combine chitosan with nanoparticles in current and future applications. For instance, chitosan can be used to modify nanoparticles; on the other hand, it can also be functionalized with nanoparticles. The composites of chitosan bonded with nanoparticles may have advantages over chitosan and the bonding materials alone. Chitosan-gold hybrid nanospheres and gold nanoparticles encapsulated with chitosan have been fabricated (Tan and Zhang, 2005; Guo et al., 2008). Because these hybrid composites are relatively new materials, their putative toxicity has yet to be determined. Similarly, biomedical applications of these materials remain to be developed: presumably cell culture systems *in vitro* may be suitably employed for assisting such developments.

## Pharmaceutical applications of chitosan and nanoparticles

Because of good biocompatibility, chitosan has excellent potential in numerous pharmaceutical applications; consequently, it has captured the attention of researchers in pharmaceutical sciences (Dodane and Vilivalam, 1998). The fact that chitosan is highly biocompatible, biodegradable, and shows the ability to open intercellular tight junctions renders it an almost ideal candidate for formulating drugs for oral delivery (Berradaa et al., 2005). For example, a chitosan-dibasic orthophosphate hydrogel has been formulated and synthesized (Ta et al., 2009). The potential of employing this gel as a prolonged drug delivery vehicle was demonstrated using fluorescein isothiocyanate-dextran, β-lactoglobulin and bovine serum albumin. Moreover, this hydrogel was not toxic to SaOS-2 (human epithelial-like osteosarcoma) cells.

Nanoparticulate delivery system has the potential to improve drug stability, increase the duration of the therapeutic effect and permit administration through non-parental routes. Sarmento et al. (2007a,b) prepared alginate/chitosan nanoparticles and dextran sulfate/chitosan nanoparticles for use in oral insulin delivery. They found that the nanoparticles significantly lowered serum glucose levels in streptozotocin-induced diabetic rats at insulin doses of 50 and 100 IU/kg. The hypoglycemic effect and insulinemia levels were considerably better than those obtained from oral insulin administration alone. Their results indicated that alginate/chitosan nanoparticles and dextran sulfate/chitosan nanoparticles showed favorable characteristics as agents to be employed for formulating oral delivery system for insulin and potentially for other therapeutical proteins. Rekha and Sharma (2009) synthesized and evaluated lauryl succinyl chitosan particles for the applications in oral insulin delivery and absorption. Their results demonstrated that the chitosan particles modified with both hydrophilic (i.e., succinyl) and hydrophobic (i.e., lauryl) moieties had improved the release characteristics, mucoadhesiveness as well as the permeability of insulin compared to those of the native chitosan particles. Thus, their findings suggested that these novel chitosan derivatives may be promising candidates for oral peptide delivery.

Chitosan has also been employed to enhance drug efficacy, especially in formulating anti-cancer drugs. Kim et al. (2008) developed self-assembled glycol chitosan (HGC) nanoparticles for the sustained and prolonged delivery of anti-angiogenic small peptide (containing the anti-angiogenic Arg-Gly-Asp (RGD) peptide) drugs in cancer therapy. They observed that intravenous and intratumoral administration of RGD-HGC nanoparticles into B16F10 tumor-bearing mice resulted in significant decreases in tumor growth and microvessel density compared with corresponding parameters in the tumor-bearing mice injected with native RGD peptide. Thus, their findings suggested the HGC nanoparticles may be valuable in anti-angiogenic therapy and for local and regional tumor therapy. HGC nanoparticles loaded with the anticancer drug docetaxel (DTX) were investigated by Hwang et al. (2008). The DTX-HGC nanoparticles exhibited higher anti-tumor efficacy as indicated by reduced tumor volume and increased survival rate in mice bearing A549 lung cancer cells. Furthermore, the formulation of DTX-HGC nanoparticles resulted in decreased toxicity of DTX in mice compared to toxicity of free DTX in tumor-bearing mice. Consequently, their findings suggested that this type of nano-sized drug carriers may be ideal for formulating drugs for cancer treatment.

A new type of drug delivery system (DDS) involved chitosan-modified single walled carbon nanotubes (SWNTs) for controllable loading/release of an anti-cancer drug, doxorubicin (DOX) (Ji et al., 2011). The DDS containing DOX could effectively kill the HepG2 SMMC-7721 cells and depress the growth of liver cancer in tumor-bearing nude mice. Thus, this finding demonstrated that the efficacy of the chitosan-containing DDS with DOX was superior to that of free DOX and suggested that the chitosan-containing DDS holds promise for delivering anti-cancer drugs because of its improved efficacy and low side effects.

## Conclusions

As a biocompatible material, chitosan has found many and diverse biomedical applications. Nevertheless, there are lots of room for improvement in mechanical properties and biocompatibility of chitosan before it becomes an ideal biomaterial for biomedical and tissue engineering applications. For instance, blending chitosan with other polymers can improve its properties and vice versa. Chitosan can also be used to modify other materials to enhance their biocompatibility. On the other hand, chitosan can induce both stimulatory and inhibitory effects on fibroblasts and several other cell types. These contrasting data could be attributed, at least in part, to dissimilar properties and chemical compositions of the biopolymers employed, thus rending it difficult to derive molecular mechanisms underlying such dissimilar effects of chitosan. By the same token, blending chitosan with other biopolymers can very well complement what chitosan cannot achieve alone biocompatibilitywise. Consequently, there is a definite need for a systematic approach to categorically classify all the aforementioned parameters.

Due to the ubiquitous existence of nanomaterials, human exposure to nanoparticles is inevitable. Nanoparticles can enter the body through lungs, skin, or via the gastrointestinal track with intake of food, drinks, and medications, or simply by direct exposure to adverse environment such as working in a dusty tunnel. These particles can affect brain, liver, kidney, heart, blood, and other organs and tissues. They are known to induce many cytotoxic effects on neural and non-neural cell types. They may distort and inhibit cell growth leading to various pathophysiological states in humans and animals. Consequently, nanotoxicology research is now becoming an important endeavor and is gaining and receiving much more attention in the biomedical field.

Before a new material can be employed for tissue engineering or other biomedical applications, it is necessary to test its biocompatibility and/or putative toxicity. Cell cultural models *in vitro* constitute convenient systems for high throughput screening of the putative toxicity of nanoparticles and other nanomaterials (Rajaraman et al., 1974; Lai et al., 2007, 2008a, 2009, 2010; Jaiswal et al., 2010, 2011; Jain et al., 2011; Lu et al., 2011). Use of these cell models is relatively inexpensive as compared to the use of animal models. The knowledge obtained from the research can lead to prevention of human exposure to nanoparticles and/or nanomaterials that are proven hazardous. Thus far, many nanoparticles show some toxicity to some cell types. Consequently, one needs to pay more attention to the cytotoxicity of nanoparticles and other nanomaterials. On the other hand, one can exploit the inhibitory effects of nanoparticles on cell survival in cancer chemotherapy and drug treatment. Nevertheless, evaluation of nanotoxicity with standard protocols is an urgent issue as more nanomaterials are being used in industry: that includes exposure to workers in confined quarters as well as to the public in an open environment. Whilst cell models are viewed to be the most viable means to pursuit nanotoxicity, the traditional discussion of the pros and cons of cell model versus animal model still persist and is beyond the scope of this review.

Biopolymer films, either based on chitosan itself or chitosan binding with other polymers, have been demonstrated to work well with various types of cells in culture: this encouraging characteristic of chitosan holds promise for a variety of applications in current and future developments of tissue engineering. However, it would be unrealistic to expect that chitosan and its derivatives would fulfill all the needs in tissue and bioengineering. Most of our organs and tissues are multifunctional; there are receptors and organelles on or beneath the surface of the tissues and cells that function interactively in networks to either promote or suppress biological activities so that our bodily functions can be properly controlled. In fact, these push-pull properties from the multifaceted observations of chitosan and its aforementioned modified forms mimic certain characteristics of tissues and organs. This biomimicry provides a foundation that researchers can build upon. From a tissue engineering point of view, scientific advances have been made in great strides in the last few decades, but the overall state of the art is still in the developmental stage as compared to the development of the electronic industry. One should expect that the development of bioengineering be at a slower rate due to the complexity and variability of the biosystems. To say the least, research needs and opportunities in tissue and bioengineering are abundant now and in the foreseeable future.

### Conflict of interest statement

The authors declare that the research was conducted in the absence of any commercial or financial relationships that could be construed as a potential conflict of interest.
